# Molecular Detection of *Mycobacterium leprae* and the Process of Infection and Illness in Contacts of Leprosy Patients: A Systematic Review

**DOI:** 10.3390/tropicalmed10020032

**Published:** 2025-01-23

**Authors:** Sarah Lamas Vidal, Lavínia Cássia Ferreira Batista, Daniele dos Santos Lages, Bruna Eduarda Bortolomai, Isabela de Caux Bueno, Eyleen Nabyla Alvarenga Niitsuma, Nathan Guilherme de Oliveira, Ida Maria Foschiani Dias Baptista, Francisco Carlos Félix Lana

**Affiliations:** 1 Postgraduate Program in Nursing, School of Nursing, Federal University of Minas Gerais, Belo Horizonte CEP 30130-100, Minas Gerais, Brazil; sarahlamasvidal@ufmg.br (S.L.V.); daniele-lages@ufmg.br (D.d.S.L.); 2 Lauro de Souza Lima Institute, Bauru CEP 17034-971, São Paulo, Brazil; lavinia.ferreira@unesp.br (L.C.F.B.); bruna.bortolomai@unesp.br (B.E.B.); ng.oliveira@unesp.br (N.G.d.O.); imf.baptista@unesp.br (I.M.F.D.B.); 3 School of Nursing, Federal University of Minas Gerais, Belo Horizonte CEP 30130-100, Minas Gerais, Brazil; isabeladecaux@gmail.com; 4 Federal Institute of Education, Science and Technology of Northern Minas Gerais, Almenara CEP 39900-000, Minas Gerais, Brazil; eyleen.alvarenga@ifnmg.edu.br; 5 Department of Maternal and Child Nursing and Public Health, School of Nursing, Federal University of Minas Gerais, Belo Horizonte CEP 30130-100, Minas Gerais, Brazil

**Keywords:** leprosy, *Mycobacterium leprae*, epidemiological monitoring, molecular epidemiology, polymerase chain reaction

## Abstract

Various techniques have been used for the molecular identification of *Mycobacterium leprae* (*M. leprae*). The aim of this review was to identify the relationship between the molecular presence of *M. leprae* and the process of infection and/or illness of contact of leprosy cases. A systematic review was carried out by searching the Medical Literature Analysis and Retrieval System Online (MEDLINE), Cochrane Library, Embase, Scopus, Web of Science and the Latin American and Caribbean Health Sciences Literature (LILACS) databases in January 2024. The studies were selected by two pairs of reviewers. Observational cross-sectional, case–control and cohort studies were included. A descriptive synthesis of the results by category was carried out. A total of 443 studies were identified, and 36 were included in the review. Twelve molecular targets were tested to identify the presence of the bacillus. A relationship was established between the identification of *M. leprae* DNA and factors related to the index case, housing characteristics, living conditions, epidemiology and anti-PGL-1 serology. None of the studies identified evaluated the molecular viability of *M. leprae* among contacts. The detection of *M. leprae* DNA alone does not necessarily predict the development of infection or clinical illness among contacts.

## 1. Introduction

The current global strategy against leprosy has a target of zero leprosy, disability and stigma by 2030 [1]. Despite this, in 2023, diagnoses of new cases in children under 15 years old and people with established physical disability were still being made in some countries, revealing the maintenance of the chain of transmission and late diagnosis [2].

The pathogenesis of leprosy initiates after the exposure of a susceptible individual to Mycobacterium leprae (*M. leprae*), which is transmitted by the upper airways and presents high infectivity and low pathogenicity. In this sense, only a portion of the population develops the disease, and the clinical manifestations are largely determined by the immunological response of the individual [3].

Leprosy is a spectral disease where the tuberculoid (T) pole [4] is characterized by a strong cellular mediated immune response against *M. leprae* [5]. In the opposing pole, the lepromatous (L) leprosy forms [4], and this occurs in highly susceptible individuals that have a weak cell immune response to the pathogen and a high bacterial load, being considered the main source of infection [5]. Between the two poles, the intermediate forms are known as borderline [4].

**Figure 1:**
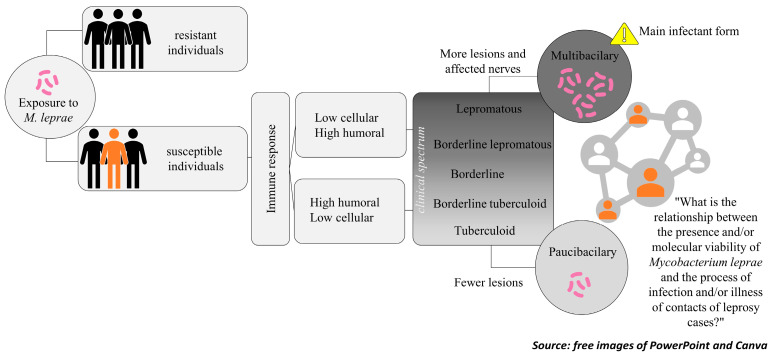
Flowchart of the pathogenesis of leprosy.

Individuals in the L pole usually present more than five skin lesions and/or a positive bacilloscopy, being classified as Multibacillary (MB) patients as per the therapeutic regimen. The individuals with less than five skin lesions and a negative bacilloscopy, that are in the T pole, are classified as Paucibacillary (PB) [3]. The main pathogenic aspects of leprosy are illustrated in Figure 1.

Close and prolonged contact with individuals with a high bacillary load favors infection by *M. leprae*. Thus, being in contact with an index case of leprosy entails a greater risk of becoming ill due to exposure to the bacillus [3].

To this day, there is no diagnostic test for the disease, which remains essentially clinical [6]. However, since the sequencing of the bacillus genome [7], advances have been made in molecular biology techniques for identifying its DNA in clinical samples.

Conventional PCR and real-time quantitative PCR (qPCR) assays [8] have been used to amplify different molecular targets of *M. leprae* and identify small numbers of bacilli. In addition, several studies have assessed viability based on RNA identification by quantitative reverse transcriptase PCR (RT-PCR) assays [9,10,11].

These techniques have been evaluated in research and recommended to support the detection of leprosy cases in field work, in light of the clinical findings [12], as aids in the diagnosis of cases for confirmation, monitoring of treatment, detection of resistance [13], as well as to help diagnose leprosy in contacts with clinical suspicion [6].

Despite the various techniques currently used for the molecular identification of *M. leprae*, as well as studies regarding people who are in contact with leprosy cases (which document their role as asymptomatic carriers of the bacillus that may contribute to the risk of infection [14]), a major gap still exists regarding the progress made in the molecular biology of *M. leprae* applied to the contacts of leprosy patients and the process of infection and illness in this group. This gap converges with the need to define a test for diagnosing the disease before clinical signs appear, and this review aims to synthesize existing knowledge in order to answer the following question: “What is the relationship between the presence and/or molecular viability of *Mycobacterium leprae* and the process of infection and/or illness of contacts of leprosy cases?”.

## 2. Materials and Methods

The reporting of this systematic review follows the Preferred Reporting Items for Systematic Reviews and Meta-Analyses (PRISMA) guidelines [15]. A protocol was drawn up and registered under the code CRD42022381295, and it is available for access on the International Prospective Register of Systematic Reviews (PROSPERO) platform, Centre for Reviews and Dissemination, York, UK.

The search strategy was defined based on specific terms designed to retrieve studies related to each of these three concepts: leprosy, the molecular presence and viability of *M. leprae*, and transmission among contacts. The terms were selected from the MeSH, DeCS and Emtree controlled vocabularies. Significant free terms were also included. No language, date/period or publication format restrictions were applied. Specific strategies were defined for the following databases: Medical Literature Analysis and Retrieval System Online (MEDLINE) via PubMed, Cochrane Library, Embase, Scopus, Web of Science and Latin American and Caribbean Health Sciences Literature (LILACS) via the regional portal of the Virtual Health Library (VHL). The complete search strategy used in each of the bases is presented in Supplementary Material Table S1. The searches were carried out on 22 January 2024. Moreover, on 9 December 2024, a new search was carried out with the official strategies on all six defined databases to verify the existence of recent articles that could attend the inclusion criteria in the present revision.

The studies retrieved from the databases were selected based on criteria that met the construction of the research question, derived from the PECOT model, in which P is the household or social contacts of patients with leprosy, E is *M. leprae* (molecular presence or viability), C is not applicable, O is infection or illness and T is published descriptive and analytical observational cross-sectional, case–control and cohort studies. Furthermore, we included articles written in Portuguese, English or Spanish, as well as articles whose original text had been translated to one of the previously described languages. Articles that could not be retrieved in full-text form, and those that did not present a description of frequency and/or effect and/of predictive measures, were not included.

The references found were exported to the Rayyan QCRI (Qatar Computing Research Institute) software, Cambridge, MA, USA. They were then added together and duplicates were excluded. The studies were then selected by reading the title and abstract by two pairs of reviewers (SLV and LCFB; DSL and BEB) independently and blinded on the Rayyan platform. Once the selection was complete, the pairs’ choices were compared and any differences were resolved by a third reviewer (ICB).

After this first stage, the selected references were entered into the Mendeley Reference Manager software, Elsevier Limited, London, United Kingdom of Great Britain and Northern Ireland, where the reviewers had access to the full text. The full-text selection was carried out by a pair of reviewers (SLV and LCFB) by filling in a spreadsheet created in Microsoft Excel and fed in by each reviewer independently. The comparison was made after the selection had been completed. An agreement between the two reviewers was assessed using the Kappa test, and substantial and significant agreement was observed (Kappa = 0.651; *p* < 0.0001). Disagreements were again resolved by a third reviewer (ENAN).

The following data were extracted from the eligible studies: authors, year of publication, journal, country, endemicity, study design, study period, population (household or social contact), sample size, comparison group (if any), outcome assessed, other variables included in the study (sex, age group, clinical form of the case, operational classification of the case, etc.), biological material analyzed, collection site, target used, analysis technique, frequency and/or effect measures assessed with confidence interval and *p*-value (if available). The information was entered into a standardized table in two independent entries and then compared to produce a summary of the review.

The methodological quality of the included studies was assessed using the Newcastle–Ottawa Scale (NOS). This scale helps to assess studies in terms of quality, assigning scores based on a star system, ranging from zero to nine stars for case–control and cohort studies and from zero to seven stars for cross-sectional studies. The studies are analyzed in relation to selection, comparability and exposure or outcome [16,17]. The results were drawn up by aggregating the data extracted into categories and then describing each category in narrative form.

## 3. Results

**Figure 2:**
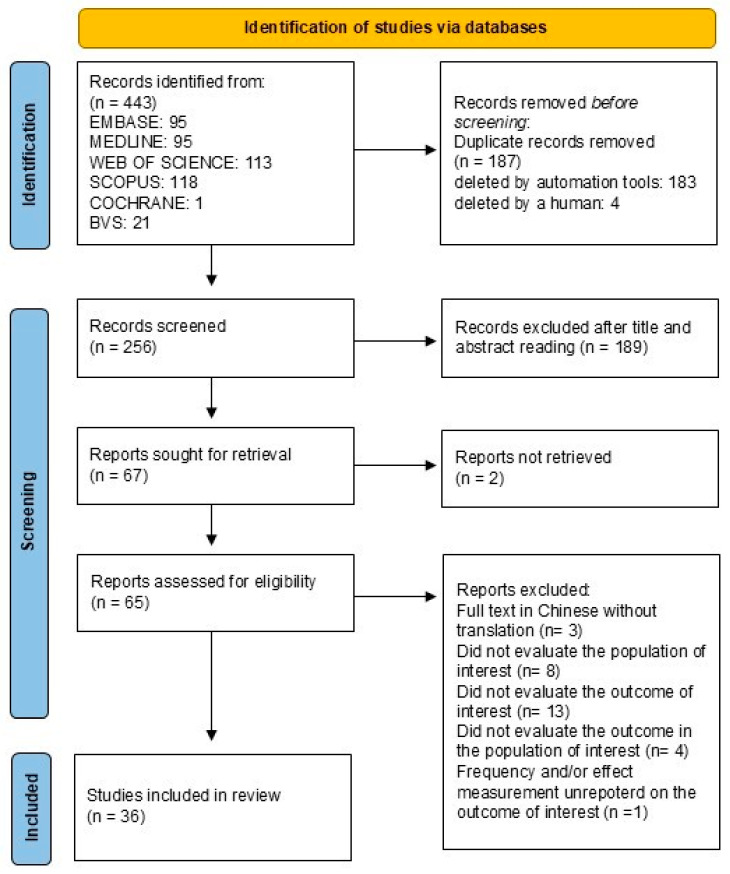
PRISMA diagram: the process of selecting eligible studies for the systematic review.

The database search resulted in 443 studies. After removing duplicates, 256 titles and abstracts were evaluated. Of these, 65 studies were selected for full-text reading, of which 29 were excluded (Supplementary Material Table S2). At the end of the selection process, detailed in Figure 2, 36 studies were considered eligible for this systematic review.

The 36 studies included were published between 1997 and 2023 and involved eight countries: Argentina, Bangladesh, Brazil, China, Colombia, Ethiopia, India and Indonesia. Most of the publications (52.8%) were carried out in Brazil. The most frequent language among the publications was English, representing 91.7% of all studies. Moreover, two studies were published in Spanish and one was published in Portuguese. With regard to study design, 24 (66.6%) were cross-sectional studies, 10 (27.8%) were cohort studies, one (2.8%) was a case–control study and one (2.8%) was a follow-up study.

Among the types of contacts evaluated, 32 studies (88.9%) evaluated household contacts, three (8.3%) evaluated household and peridomiciliary contacts, and only one study (2.8%) evaluated social contacts, these being schoolchildren. The sample ranged from 18 to 1352.

The methodological quality of the studies was assessed using the NOS. In the cross-sectional studies, the score ranged from two to six stars (maximum seven), with 13 (54.2%) receiving four stars. In the cohort, follow-up and case–control studies, the score ranged from four to eight stars (maximum nine), with the majority (66.7%) achieving five or six stars. The main limitations that negatively impacted the score of the studies included lack of comparability between groups, an insufficient follow-up period and losses during follow-up (Supplementary Material Table S3).

The data extracted from the molecular analyses and the outcomes assessed (Supplementary Material Table S4) were grouped and presented in three categories.

Category 1—*M. leprae* positivity in contacts

All 36 studies presented results regarding the presence of *M. leprae* among contacts. The majority of studies (n = 23–63.9%) assessed the presence of the bacillus using the RLEP sequence [14,18,19,20,21,22,23,24,25,26,27,28,29,30,31,32,33,34,35,36,37,38,39]. Another five studies (13.9%) identified the 16S rRNA target [37,38,40,41,42], while less frequent targets, such as the *pra* [43,44], the 12-5 sequence [45], 36 kDa [46], the sequence LP1 (5′-TGCATGTCATGGCCTTGAGG-3′) and LP2 (5′-CACCGATACCAGCGGCAGAA-3′) [47], LSR/A15 [48], S13/S62 [49], 18 kDa [50], ML0024 [51], *sodA* [38] and 85B [39] were also evaluated. One of the studies did not identify which molecular target was used for the investigation [52]. It should be noted that three studies evaluated more than one molecular target in their investigation [37,38,39].

1.1. Positivity of *M. leprae* from the RLEP sequence

The prevalence of positivity for *M. leprae* DNA from the RLEP sequence varied widely. The conventional PCR technique had a positivity of 1.7% [21] to 72.2% [27] for nasal swabs, 5.6% [29] to 6,83% [30] for oral swabs, 1.7% [21] to 6.25% [20] for blood samples and 3.12% [19] to 21.43% [18] in dermal scrapings. When the qPCR technique was performed, positivity for nasal swab samples ranged from 4.7% [35] to 49% [14], while, for dermal scrapings, it ranged from 23.7% [32] to 78.7% [34]. In palate scraping, it was 31% [39].

Among the studies that tested more than one type of sample for RLEP, the percentage of positivity observed was similar between blood (1.7%) and nasal swabs (1.7%) [21], between oral swabs (5.6%) and nasal swabs (4.6%) [29] and between earlobe dermal scrapings (12.3%) and nasal swabs (18.0%) [31]. One study compared three types of samples and found a small difference between nasal swab (49%) and nasal biopsy (53.8%), but these results differed by more than 40% from the positivity found in blood samples (6.7%) [14].

1.2. Positivity of *M. leprae* from the 16S target

Three studies have evaluated the presence of *M. leprae* using the 16S rRNA target [40,41,42]. All used the qPCR technique and evaluated dermal scraping samples. One of the studies also evaluated blood samples and found a positivity rate of 9.73% [40]. In dermal scrapings, positivity ranged from 9% [42] to 16.81% [41].

One of these studies evaluated the molecular presence of the bacillus in three different years, observing a progressive decrease in the positivity from 16.81% to 7.04% in the following year and, four years later, to 0%. It is important to note that the number of contacts assessed also decreased over the course of the follow-up process [41].

1.3. Positivity of *M. leprae* from different molecular targets

Among the studies that evaluated other targets, less frequently than those described above, eight performed conventional PCR, and the prevalence of positivity to *M. leprae* ranged from 0.6% with evaluation of the *pra* gene to [44] to 31% with the 12-5 sequence [45].

Of these, only two studies carried out more than one molecular assessment among contacts, allowing for the presence of *M. leprae* DNA in these individuals to be monitored. One of them evaluated three subsequent months following the treatment of the index case and identified a reduction in the presence of the bacillus among contacts in both nasal swabs and scrapings, with the results showing 0% bacillary DNA among contacts after two months of treatment of the case [50]. Another study, which carried out an initial assessment and a second one after one year, identified an increase in the prevalence of positivity among contacts in nasal mucosa. However, there were losses during follow-up, and, despite the increase in the percentage of positive results, this positivity was not maintained among the same individuals, indicating that positivity is transitory [49].

In two other studies that used the qPCR technique, the prevalence varied from 1.2% for the ML0024 target in blood samples [51] to 19.35% in nasal biopsies [52] without specifying the target used.

1.4. Positivity of *M. leprae* from more than one molecular target

Three studies evaluated the prevalence of *M. leprae* DNA positivity using more than one molecular target. In all of them, the RLEP was evaluated and showed the highest percentages of positivity [37,38,39]. One study compared the positivity percentage in the saliva of households and peridomiciliary contacts for the RLEP and 16S targets, finding greater positivity among peridomiciliary contacts than among those who lived in the same household as the leprosy case [37].

Two other studies evaluated only household contacts. One of them compared the positivity of nasal swabs and palate scrapings for the RLEP and 85B targets and found no significant difference between the two collection sites, indicating that both the nasal mucosa and the oral cavity are equally colonized by the bacillus [39]. Another study evaluated the RLEP, 16s and *sodA* targets in blood samples, nasal swabs, earlobe dermal scrapings and saliva, identifying positivity only in nasal swabs. Among the molecular targets, the highest positivity was for RLEP (10%), followed by 16S (4%), while *sodA* showed no positivity. When the three targets were evaluated in a single reaction using multiplex PCR, positivity increased considerably to 40% [38].

In Table 1, the data extracted in the present study regarding the *M. leprae* positivity in contacts for the RLEP sequence, 16S target and other molecular targets is summarized.

Category 2—Presence of *M. leprae* and associated factors

Some studies have found an association between the prevalence of positivity to *M. leprae* and factors related to the index case’s [34] housing characteristics, socializing and epidemiological aspects [36,47,48], as well as positive anti-PGL-1 serology [14,31,35].

It was found that contacts of cases classified as BL and LL were more likely to detect *M. leprae* than contacts of TT cases (OR: 6.6; 95% CI: 1.6–27.6; *p* = 0.0090). Similarly, contacts of MB cases (OR: 4.88; 95% CI: 1.02–23.37; *p* = 0.04) and those with a positive baciloscopic index (BI) (OR: 7.07; 95% CI: 1.41–35.41; *p* = 0.0173) also showed greater molecular positivity. In addition, being a genetic contact of the index case further increased the chances of positivity when the case was LL or BL (OR: 9.23; 95% CI: 1.01–83.94; *p* = 0.04) and had a positive BI (OR: 6.93; 95% CI: 0.76–63.04; *p* = 0.08) [34].

When evaluating living with the index case and housing and socioeconomic conditions, it was observed that contacts who had lived with the index case for more than a year were more likely to have the bacillus in their body (OR: 12.45; 95% CI: 1.595–97.20; *p* = 0.002) [47]. In addition, spouses, regardless of the degree of kinship, also had a higher chance of infection compared to other household contacts (OR: 3.87; 95% CI: 1.21–12.3) [48].

With regard to socioeconomic conditions, a study carried out with social contacts found that living in regions with a poor socioeconomic situation (prevalence ratio (PR): 0.40; 95% CI: 0.18–0.91; *p* = 0.039) and living in rented or donated property (PR: 2.16; 95% CI: 1.16–4.04; *p* = 0.015) are associated with a greater chance of infection with *M. leprae* [36].

Regarding the association with serology, one study found that individuals who had *M. leprae* in nasal biopsy samples also had an immune response identified by anti-PGL-1 serology compared to the others (OR: 4.2; 95% CI: 1.2–14.8; *p* = 0.046) [14]. The same association was confirmed in another study which evaluated the detection of *M. leprae* DNA in nasal swabs (*p* < 0.0034) [35]. Furthermore, the negative correlation identified between RLEP Ct values and serum anti-PGL-1 levels in nasal swab and earlobe dermal scraping samples corroborates this relationship between the presence of bacillus DNA and antibody expression [31].

Category 3—Presence of *M. leprae* and illness among contacts

Three studies analyzed the association between the identification of *M. leprae* in molecular tests and the illness in contacts. One of them evaluated the presence of the bacillus in the blood and identified a 14 times greater chance of becoming ill in relation to those who were not positive in the molecular evaluation (OR = 14.78; 95% CI: 3.6–60.8; *p* < 0.0001) as well as a positive likelihood ratio (LR+) = 13.19 (95% CI: 3.6–48.1; *p* < 0.0001) [51]. Another study found an association between the development of leprosy and positive results in blood samples, with Relative Risk (RR) and LR+ = 5.54 (95% CI: 1.30–23.62) [14]. The third study investigated the association between the positivity of *M. leprae* present in dermal scrapings from earlobes and becoming ill, but the RR of 2.52 (95% CI: 0.28–22.35) was not statistically significant [42].

In addition to these three, ten other studies [19,21,22,23,26,31,32,40,41,52] identify the occurrence or absence of illness among contacts, but they do not establish a direct relationship with the positivity identified in the molecular tests.

Among the thirteen studies, the highest percentage of illness identified among contacts was 11.8% [23], while the lowest was 0% among contacts assessed during a one-year follow-up [21].

## 4. Discussion

From the systematic review, it was identified that (a) there is a great diversity of molecular targets already tested to identify *M. leprae* DNA in contacts; (b) none of the studies evaluated investigated the molecular viability of *M. leprae* among contacts; and (c) the studies analyzed do not provide sufficient evidence to correlate the identification of bacillus DNA by a specific molecular target alone with infection and the development of leprosy in contacts.

Twelve molecular targets were evaluated among the studies included in this review: RLEP, 16S rRNA, pra gene, 12-5 sequence, 36 kDa, sequence LP1 (5′-TGCATGTCATGGCCTTGAGG-3′) and LP2 (5′-CACCGATACCAGCGGCAGAA-3′), LSR/A15, S13/S62, 18 kDa, ML0024, sodA and 85B. Of the 36 studies, 63.9% used the RLEP sequence. It also had the highest percentage of positives identified. The high proportion of studies using this sequence may be related to the number of copies of the sequence in the bacillus genome [53], making it a widely used target.

Although numerous studies have shown that both the sensitivity and specificity of qPCR are superior to those of conventional PCR [8,54], in this review it was not possible to define greater or lesser detections between the two techniques due to the variations in positivity results for *M. leprae* DNA identified in both techniques. However, qPCR enables direct quantification of the DNA content in clinical samples, considerably improving the time taken to obtain the result [13]. Among the studies evaluated in this review, qPCR has been more widely used in recent years.

There was also great variability in relation to the biological material analyzed, regardless of the technique used. The efficiency of PCR can be linked to numerous factors other than the type of sample, such as those related to the technique used to collect the material, possible contamination of the samples, pipetting faults [55] and correct determination of the fluorescence threshold [56], among other aspects that can affect the result of the molecular evaluation.

Factors such as being a contact of a case classified as having the most severe form of leprosy and living with the index case for longer and being their spouse increased the chance of the contact having bacillus DNA. It is known that people who develop the most severe forms of the disease have a greater amount of bacillus in their bodies [6]. In addition, living conditions interfere with the chance of infection by *M. leprae* due to exposure to the bacillus.

In social contacts, a relationship was observed between the presence of bacillary DNA and poor housing conditions. Leprosy is consistently related to unfavorable socioeconomic conditions [57,58], such as living in areas of greater poverty [58]. Leprosy is a disease historically linked to the individual’s social context, which involves both individual and collective aspects [59]. Factors such as low levels of schooling, food insecurity [57,59], being brown or black [58], living with a case for more than five years, living in the same house as the index case, having overlapping cases in the family [60] and living with an index case with a high bacillary load [61] are in line with the findings of the studies included in this review which show a relationship between unfavorable socioeconomic factors and the identification of *M. leprae* DNA among contacts.

Relationships have also been established between the presence of *M. leprae* DNA and anti-PGL-1 serology. Serological tests are known to be useful in identifying infected individuals in endemic areas [62] and non-endemic areas [63] and can be used to screen and identify people at high risk of becoming ill [64]. Contacts who, as well as having *M. leprae* DNA, already express antibodies to the bacillus, have greater evidence of established infection and a limited cellular immune response.

The immunological aspects of the person infected by *M. leprae* play an important role in the development of leprosy and, in the manifestation of clinical signs [65], are taken into account when classifying the disease [4,66]. The time between infection and diagnosis favors bacterial proliferation and can lead to the development of physical deformities, aggravating the stigma associated with leprosy [67].

Among the studies included in this review, 25 (69.5%) concluded that molecular identification of *M. leprae* DNA indicates infection in these individuals. In the studies that did not link the presence of the bacillus to infection, the results showed that molecular identification allows us to draw conclusions about the colonization of contacts and their role in exposure and transporting of the bacillus.

Despite the high percentage (69.5%) of articles included in this review that concluded that individuals with bacillus DNA in their bodies were infected, it is important to note that some of them evaluated samples from the upper airways using nasal and oral swabs. These sites are considered to be the bacillus main entry point into the body [14], indicating bacillus carriers [68]. This may be linked to the colonization process in which the infectious agent is present without clinical manifestation or immune response.

However, among those studies that evaluated blood samples, biopsies or dermal scrapings, the possibility of infection can be considered, since the bacilli were successful in the colonization process, overcoming immunological barriers [14]. In light of those studies, we believe that infection is a possible conclusion, considering that a link is made between the presence of signs and symptoms of leprosy or positivity anti-PGL-1 and the outcome infection, which is corroborated by immunological responses.

Regarding illness in contacts, results were presented in 13 studies, but only 3 of them established a relationship with *M. leprae* DNA positivity. One of them evaluated earlobe dermal scraping samples and showed no statistical significance. Two others evaluated blood samples.

One of these studies established that the bacillus is identified in the blood of individuals who have had a successful nasal infection process, thus continuing a productive infection and spreading to places where its growth is favored [14]. The association of the outcome illness and bacillus DNA in blood rather than earlobe scrapings may be related to the technique, since blood collection is more accessible and does not require specialized training [55].

It is important to note that most of the studies included in this review had a cross-sectional design, which may have made it impossible to relate the presence of *M. leprae* DNA to the appearance of clinical signs, immune response and the development of the disease.

Considering the definition of infection as the “penetration, multiplication and/or development of an infectious agent in a given host”, which can also be defined as subclinical infection when it does not cause clinical manifestations [69], the data obtained in this systematic review make it possible to define that the identification of *M. leprae* DNA in biological samples from leprosy contacts alone does not directly correlate with infection by the bacillus. For this to happen, factors such as the presence of an immune response and the molecular viability of these bacilli must be taken into account.

Likewise, this relationship cannot be made regarding the outcome illness. Although two studies demonstrated a significant association between the presence of bacillus DNA in blood samples and a greater chance of having leprosy, the use of two different molecular targets limits the definition of that as evidence.

The detection of *M. leprae* DNA in clinical samples is important and has been recommended among the surveillance actions of the Unified Health System (SUS) since 2022; however, its use is restricted to specialized care in the skin biopsies of contacts already suspected of the disease with inconclusive alterations [6].

In addition to the identification of bacillus DNA, detecting *M. leprae* RNA by means of RT-PCR allows for the viability of the bacillus to be assessed, which has already been determined in studies of leprosy cases [9,11] and in animal models [10], and it is also suggested for assessing bacterial load [11] and identifying the process of infection and illness [9].

It is believed to be important to evaluate the molecular viability of *M. leprae* among leprosy contacts, including those without alterations suggestive of the disease, in order to verify its applicability as an early diagnostic tool. Research evaluating bacillus viability targets in household and social contacts from places with different endemicities, as well as in different biological samples, could help define the best model for identifying contacts at greater risk of developing leprosy.

It should be noted that, although the search strategy was designed to retrieve a wide range of articles, none of the studies identified assessed the viability of *M. leprae* among contacts, which is a limitation of this review. Another limitation refers to the low number of longitudinal studies identified, which highlights the difficulty of following-up contacts of leprosy cases, making it difficult to advance knowledge about the process of infection and becoming ill with leprosy. In this context, the weaknesses of the health services, especially Primary Health Care, in carrying out leprosy control actions also stand out [70], and these actions involve the monitoring and surveillance of contacts.

Our results provide insights, since they indicate a demand for new studies, especially those that evaluate molecular viability using RNA samples, thus allowing for the expansion of knowledge regarding the exact relationship between molecular targets and the development of infection and illness.

We conclude that the detection of *M. leprae* DNA per se does not provide sufficient evidence to link molecular positivity to the development of infection or illness among leprosy contacts.

## Figures and Tables

**Table 1:** *M. leprae* DNA positivity among the studies included in the systematic review.

Molecular Target	Analysis Technique	Biological Material Analyzed	*M. leprae* DNA Positivity	Reference
RLEP	PCR	Dermal scrapings	3.12% to 21.43%	[18,19,31]
		Blood	1.7% to 6.25%	[20,21]
		Nasal swab	1.7% to 72.2%	[19,21,22,23,24,25,26,27,28,29,31,38]
		Oral swab	5.6% a 6.83%	[29,30]
		Saliva	4.9% in household contacts and 12.5% peridomiciliary contacts	[37]
	qPCR	Nasal biposy	53.8%	[14]
		Dermal scrapings	23.7% to 78.7%	[32,33,34]
		Nasal swab	4.7% to 49%	[14,35,36,39]
		Blood	6.7%	[14]
		Palate scrapings	31%	[39]
16S	PCR	Saliva	An amount of 2.4% in household contacts and 2.5% in peridomiciliary contacts	[37]
		Nasal swab	4%	[38]
	qPCR	Dermal scrapings	9% to 16.81%	[40,41,42]
		Blood	9.73%	[40]
Other molecular targets	PCR	Nasal swab	0% to 31%	[38,43,44,45,46,47,48,49,50]
		Dermal scrapings	17.2%	[50]
	qPCR	Nasal swab	19%	[39]
		Palate scrapings	13%	[39]
		Blood	1.2%	[51]
		Nasal swab	19.35%	[52]

## Data Availability

The original contributions presented in the study are included in the article/Supplementary Material; further inquiries can be directed to the corresponding author.

## Supplementary Materials

The following supporting information can be downloaded at: https://www.mdpi.com/article/10.3390/tropicalmed10020032/s1. Supplementary Material Table S1—Database search strategy. Supplementary Material Table S2—Studies excluded after reading the full text. Supplementary Material Table S3—General characteristics of the included studies. Supplementary Material Table S4—Characteristics of molecular analyses and outcome.
