# Supplementary material for: Molecular Detection of Mycobacterium leprae and the Process of Infection and Illness in Contacts of Leprosy Patients: A Systematic Review
Source: Trop Med Infect Dis. 2025 Jan 23;10(2):32. doi: 10.3390/tropicalmed10020032 (PMC11860987; doi:10.3390/tropicalmed10020032)
Supplement: Supplementary file 1 [file tropicalmed-10-00032-s001.zip › tropicalmed-3296835-supplementary.pdf]

Supplementary Material Table S1 - Database search strategy

Medline - by PubMed
((("Leprosy"[MeSH Terms] OR "Hansen's Disease"[Title/Abstract] OR "Hansen Disease"[Title/Abstract] OR "leprosy*"[Title/Abstract] OR "hansen*"[Title/Abstract] OR "Mycobacterium leprae"[MeSH Terms] OR "Mycobacterium leprae"[Title/Abstract]) AND ("molecular epidemiology"[MeSH Terms] OR "molecular epidemiology"[Title/Abstract] OR "molecular diagnostic techniques"[MeSH Terms] OR "molecular diagnostic techniques"[Title/Abstract] OR "asymptomatic infections"[MeSH Terms] OR "asymptomatic infections"[Title/Abstract] OR "asymptomatic infections"[MeSH Terms] OR "subclinical infection"[Title/Abstract] OR "nasal mucosa"[MeSH Terms] OR "nasal mucosa"[Title/Abstract] OR "mouth mucosa"[MeSH Terms] OR "mouth mucosa"[Title/Abstract] OR "rna ribosomal 16s"[Title/Abstract] OR "Qpcr"[Title/Abstract] OR "RLEP"[Title/Abstract] OR "ARNr 16s"[Title/Abstract] OR "16s RNA"[Title/Abstract] OR "16Srna"[Title/Abstract] OR "PCR"[Title/Abstract] OR "Polymerase Chain Reaction"[Title/Abstract] OR "Rrna"[Title/Abstract] OR "dna bacterial"[Title/Abstract] OR "Real-Time Polymerase Chain Reaction"[Title/Abstract] OR "DNA determination"[Title/Abstract] OR "DNA extraction"[Title/Abstract] OR "DNA sequence"[Title/Abstract])) AND ("disease transmission, infectious"[MeSH Terms] OR "disease transmission infectious"[Title/Abstract] OR "Contact Tracing"[MeSH Terms] OR "Contact Tracing"[Title/Abstract] OR "family characteristics"[MeSH Terms] OR "family characteristics"[Title/Abstract] OR "Infectious Disease Transmission"[Title/Abstract] OR "Household Contact"[Title/Abstract] OR "Household Contacts"[Title/Abstract] OR "Patient Contact"[Title/Abstract] OR "Patient Contacts"[Title/Abstract] OR "Peridomiciliary Contacts"[Title/Abstract]))
**Cochrane Library**
(Leprosy OR "Hansen's Disease" OR "Hansen Disease" OR Leprosy* OR Hansen* OR "Mycobacterium leprae" OR "Mycobacterium leprae"):ti,ab,kw AND ("Molecular Epidemiology" OR "Molecular Diagnostic Techniques" OR "Asymptomatic Infections" OR "Subclinical Infection" OR "Nasal Mucosa" OR "Mouth Mucosa" OR "RNA, Ribosomal, 16S" OR "Qpcr" OR "RLEP" OR "ARNr 16s" OR "16s RNA" OR "16Srna" OR "PCR" OR "Polymerase Chain Reaction" OR "Rrna" OR "DNA, bacterial" OR "Real-Time Polymerase Chain Reaction" OR "DNA determination" OR "DNA extraction" OR "DNA sequence"):ti,ab,kw AND ("Contact Tracing" OR "Family Characteristics" OR "Infectious Disease Transmission" OR "Household Contact" OR "Household Contacts" OR "Patient Contact" OR "Patient Contacts" OR "Peridomiciliary Contacts"):ti,ab,kw
**EMBASE – by CAPES Journal Portal**
('leprosy'/exp OR 'mycobacterium leprae'/exp OR leprosy* OR 'hansen s disease' OR 'hansen disease'/exp OR 'hansen disease' OR hansen* OR 'mycobacterium leprae'/exp OR 'mycobacterium leprae' OR leprosy*:ab,ti OR 'hansen s disease':ab,ti OR 'hansen disease':ab,ti OR hansen*:ab,ti OR 'mycobacterium leprae':ab,ti) AND ('molecular epidemiology'/exp OR 'molecular diagnosis'/exp OR 'asymptomatic infection'/exp OR 'nasal mucosa'/exp OR 'mouth mucosa'/exp OR 'molecular epidemiology'/exp OR 'molecular epidemiology' OR 'molecular diagnostic techniques'/exp OR 'molecular diagnostic techniques' OR 'asymptomatic infections'/exp OR 'asymptomatic infections'
Continue

Supplementary Material Table S1 - Continued

OR 'subclinical infection'/exp OR 'subclinical infection' OR 'nasal mucosa'/exp OR 'nasal mucosa' OR 'mouth mucosa'/exp OR 'mouth mucosa' OR 'rna, ribosomal, 16s'/exp OR 'rna, ribosomal, 16s' OR 'qpcr'/exp OR 'qpcr' OR 'rlep' OR 'arnr 16s' OR '16s rna'/exp OR '16s rna' OR '16srna' OR 'pcr'/exp OR 'pcr' OR 'polymerase chain reaction'/exp OR 'polymerase chain reaction' OR 'rrna'/exp OR 'rrna' OR 'dna, bacterial'/exp OR 'dna, bacterial' OR 'real-time polymerase chain reaction'/exp OR 'real-time polymerase chain reaction' OR 'dna determination'/exp OR 'dna determination' OR 'dna extraction'/exp OR 'dna extraction' OR 'dna sequence'/exp OR 'dna sequence') AND ('contact tracing'/exp OR 'contact tracing' OR 'family characteristics'/exp OR 'family characteristics' OR 'infectious disease transmission'/exp OR 'infectious disease transmission' OR 'household contact'/exp OR 'household contact' OR 'household contacts' OR 'patient contact' OR 'patient contacts' OR 'peridomiciliary contacts' OR 'contact tracing':ab,ti OR 'family characteristics':ab,ti OR 'infectious disease transmission':ab,ti OR 'household contact':ab,ti OR 'household contacts':ab,ti OR 'patient contact':ab,ti OR 'patient contacts':ab,ti OR 'peridomiciliary contacts':ab,ti OR 'disease transmission'/exp OR 'contact examination'/exp OR 'family structure'/exp) AND [embase]/lim NOT ([embase]/lim AND [medline]/lim)
**SCOPUS – by CAPES Journal Portal**
(Leprosy* OR "Hansen's Disease" OR "Hansen Disease" OR Hansen* OR "Mycobacterium leprae") AND ("Molecular Epidemiology" OR "Molecular Diagnostic Techniques" OR "Asymptomatic Infections" OR "Subclinical Infection" OR "Nasal Mucosa" OR "Mouth Mucosa" OR "RNA, Ribosomal, 16S" OR "Qpcr" OR "RLEP" OR "ARNr 16s" OR "16s RNA" OR "16Srna" OR "PCR" OR "Polymerase Chain Reaction" OR "Rrna" OR "DNA, bacterial" OR "Real-Time Polymerase Chain Reaction" OR "DNA determination" OR "DNA extraction" OR "DNA sequence") AND ("Contact Tracing" OR "Family Characteristics" OR "Infectious Disease Transmission" OR "Household Contact" OR "Household Contacts" OR "Patient Contact" OR "Patient Contacts" OR "Peridomiciliary Contacts")
**WEB of SCIENCE – by CAPES Journal Portal**
(Leprosy* OR "Hansen's Disease" OR "Hansen Disease" OR Hansen* OR "Mycobacterium leprae") AND ("Molecular Epidemiology" OR "Molecular Diagnostic Techniques" OR "Asymptomatic Infections" OR "Subclinical Infection" OR "Nasal Mucosa" OR "Mouth Mucosa" OR "RNA, Ribosomal, 16S" OR "Qpcr" OR "RLEP" OR "ARNr 16s" OR "16s RNA" OR "16Srna" OR "PCR" OR "Polymerase Chain Reaction" OR "Rrna" OR "DNA, bacterial" OR "Real-Time Polymerase Chain Reaction" OR "DNA determination" OR "DNA extraction" OR "DNA sequence") AND ("Contact Tracing" OR "Family Characteristics" OR "Infectious Disease Transmission" OR "Household Contact" OR "Household Contacts" OR "Patient Contact" OR "Patient Contacts" OR "Peridomiciliary Contacts")
**LILACS – by regional BVS portal**
((mh:Leprosy OR "Hansen's Disease" OR "Hansen Disease" OR Leprosy* OR Hansen* OR mh:"Mycobacterium leprae" OR "Mycobacterium leprae" OR Hanseníase OR lepra OR "Doença de Hansen" OR "Bacilo de Hansen") AND (mh:"Molecular Epidemiology" OR "Molecular Epidemiology" OR mh:"Molecular Diagnostic Techniques" OR "Molecular Diagnostic Techniques" OR mh:"Asymptomatic Infections" OR "Asymptomatic Infections" OR mh:"Subclinical Infection" OR

Continue

Supplementary Material Table S1 - Continued

"Subclinical Infection" OR mh:"Nasal Mucosa" OR "Nasal Mucosa" OR "Mucosa Nasal" OR mh:"Mouth Mucosa" OR "Mouth Mucosa" OR "Mucosa Bucal" OR "Mucosa Oral" OR "Mucosa da Boca" OR "RNA, Ribosomal, 16S" OR "Qpcr" OR "RLEP" OR "ARNr 16s" OR "16s RNA" OR "16Srna" OR "PCR" OR "Polymerase Chain Reaction" OR "Rrna" OR "DNA, bacterial" OR "Real-Time Polymerase Chain Reaction" OR "DNA determination" OR "DNA extraction" OR "DNA sequence" OR "Epidemiologia Molecular" OR "Técnicas de Diagnóstico Molecular" OR "Infecções Assintomáticas" OR "Colonização Assintomática" OR "Infecções Inaparentes" OR "Infecções Pré-Sintomáticas" OR "Infecções Subclínicas" OR "RNA Ribossômico 16S" OR "16S rRNA" OR "DNA Bacteriano")) AND (mh:"Disease Transmission, Infectious" OR "Disease Transmission, Infectious" OR mh:"Contact Tracing" OR "Contact Tracing" OR mh:"Family Characteristics" OR "Family Characteristics" OR "Infectious Disease Transmission" OR "Household Contact" OR "Household Contacts" OR "Patient Contact" OR "Patient Contacts" OR "Peridomiciliary Contacts" OR "Transmissão de Doença Infecciosa" OR "investigação de Contato" OR "Notificação do Parceiro" OR "Triagem de Contato" OR "Contato Domiciliar" OR "Contatos Domiciliares" OR "Contato do Paciente" OR "Contatos do Paciente" OR "Contatos Peridomiciliares") AND (db:("LILACS"))

Supplementary Material Table S2 - Studies excluded after reading the full text

Titles	Reason for exclusion
An epidemiological study of leprosy infection by serology and polymerase chain reaction	Does not evaluate the outcome of interest
Blood RNA signature RISK4LEP predicts leprosy years before clinical onset	Does not evaluate the outcome of interest
Clinical manifestations of leprosy after BCG vaccination: An observational study in Bangladesh	Does not evaluate the outcome of interest
Development of a combined RLEP/16S rRNA (RT) qPCR assay for the detection of viable *M. leprae* from nasal swab samples	Does not evaluate the population of interest
Dynamics of Mycobacterium leprae transmission in environmental context: Deciphering the role of environment as a potential reservoir	Does not evaluate the outcome in the population of interest
Early diagnosis of neural impairment in seropositive leprosy household contacts: The experience of a reference center in Brazil	Does not evaluate the outcome of interest
Evaluation of PCR mediated DNA amplification in noninvasive biological specimens for subclinical detection of Mycobacterium leprae	Does not evaluate the population of interest
Frecuencia de Infección por Mycobacterium Leprae en convivientes de pacientes con lepra, Antioquia 2001-2002	Absence of a measure of frequency and/or effect related to the outcome of interest
Genotyping of Mycobacterium leprae in Myanmar and supposed transmission mode	Does not evaluate the outcome of interest
Genotyping of Mycobacterium leprae on the basis of the polymorphism of TTC repeats for analysis of leprosy transmission.	Does not evaluate the outcome of interest
Household Contacts of Leprosy Patients in Endemic Areas Display a Specific Innate Immunity Profile	Does not evaluate the outcome in the population of interest
Immunoepidemiological studies on subclinical infection among leprosy household contacts in Thailand	Does not evaluate the outcome of interest
Leprosy among schoolchildren in the Amazon region: A cross-sectional study of active search and possible source of infection by contact tracing	Does not evaluate the outcome in the population of interest
Leprosy in a prison population: A new active search strategy and a prospective clinical analysis	Does not evaluate the population of interest
Leprosy transmission in endemic and non-endemic areas based on the profile of antibody response of PGL-1 and PCR detection of Mycobacterium leprae DNA from nasal swab among healthy children of East Java, Indonesia	Does not evaluate the population of interest
Molecular, immunological and neurophysiological evaluations for early diagnosis of neural impairment in seropositive leprosy household contacts	Does not evaluate the outcome of interest

Continue

Titles	Reason for exclusion
Polymerase chain reaction for detection of Mycobacterium leprae in nasal swab specimens	Does not evaluate the population of interest
Preliminary study on leprosy subclinical infection and its sero-epidemiology	Full text in Chinese, no translation
Risk factors for developing leprosy - A population-based cohort study in Indonesia	Does not evaluate the outcome of interest
RLEP LAMP for the laboratory confirmation of leprosy: towards a point-of-care test	Does not evaluate the outcome of interest
Seroepidemiologic survey of the household contacts of leprosy patients	Does not evaluate the outcome of interest
Serological Immunoassay for Hansen's Disease Diagnosis and Monitoring Treatment: Anti-Mce1A Antibody Response Among Hansen's Disease Patients and Their Household Contacts in Northeastern Brazil	Does not evaluate the outcome in the population of interest
Spatial distribution pattern of new leprosy cases under 15 years of age and their contacts in Sobral, Ceará, Brazil	Does not evaluate the outcome of interest
Standardization of SYBR Green-Based Real-Time PCR Through the Evaluation of Different Thresholds for Different Skin Layers: An Accuracy Study and Track of the Transmission Potential of Multibacillary and Paucibacillary Leprosy Patients	Does not evaluate the population of interest
Study on subclinical infection with *M. leprae*--a follow-up	Full text in Chinese, no translation
Study on the factors influencing steady transmission of leprosy in Qiubei county, China	Full text in Chinese, no translation
The determinants of the geographical distribution and transmission of 16S rRNA of M.leprae in endemic areas, Indonesia	Does not evaluate the population of interest
The epidemiology of leprosy in a high prevalence village in Papua New Guinea.	Does not evaluate the outcome of interest
Widespread nasal carriage of Mycobacterium leprae among a healthy population in a hyperendemic region of northeastern Brazil	Does not evaluate the population of interest

Supplementary Material Table S3 - General characteristics of the included studies

Citation	Year of publication	Location (Country)	Study design	Sample - Type of Contact (n)	Other groups (n)	NOS*
Turankar et al	2014	India	Cross-sectional	Household (28)	Cases (n=5)	3
Cardona-Castro et al	2009	Colombia	Follow-up	Household (32)	Cases (n=12)	6
Wen et al	2013	China	Cross-sectional	Household (96)	Cases (n=79), Healthy control (n=35), Tuberculosis patients (n=18).	4
Almeida et al	2004	Brazil	Cohort	Household (125), and of these 120 underwent molecular evaluation	.	6
Banerjee et al	2010	India	Cohort	Household (182)	Cases (n=439)	5
Barreto	2011	Brazil	Cross-sectional	Household (187), and of these 120 underwent molecular evaluation	Cases (n=46)	4
Lourenço et al	2017	Brazil	Cross-sectional	Household (101), and of these 87 underwent molecular evaluation	Cases (n=69)	4
Medina et al	2010	Argentina	Cross-sectional	Household (26)	.	3

*Newcastle-Ottawa Scale

Continue

Citation	Year of publication	Location (Country)	Study design	Sample - Type of Contact (n)	Other groups (n)	NOS*
Romero-Montoya et al	2017	Colombia	Cross-sectional	Household (543), and of these 180 participated in the cohort and 113 underwent molecular evaluation	Cases (n=170), and of these 46 participated in the cohort	5
Silva et al	2017	Brazil	Cross-sectional	Household (18)	Cases (n=39)	3
Urgesa et al	2021	Ethiopia	Cross-sectional	Household (44)	Cases (n=11), Endemic control (n=44)	4
Pinho et al	2015	Brazil	Cross-sectional	Household and peridomestic (808)	Cases (n=200)	5
Martinez et al	2011	Brazil	Cross-sectional	Household (1288)	Cases (n=334)	4
Tió-Coma et al	2020	Bangladesh	Cohort	Household (250)	Cases (31)	6
Araujo et al	2016	Brazil	Cohort	Household (104)	Cases (n=113)	7
Bouth et al	2023	Brazil	Cross-sectional	Household (78)	Cases treated (n=23), Schoolchildren (178), Spontaneous demand (6)	4
da Silva et al	2021	Brazil	Cross-sectional	Household (296)	Newly diagnosed cases (n=87), treated cases (n=52), healthy individuals for endemic control (n=31).	4

*Newcastle-Ottawa Scale

Continue

Citation	Year of publication	Location (Country)	Study design	Sample - Type of Contact (n)	Other groups (n)	NOS*
Das et al	2020	India	Cross-sectional	Household (61)	Cases (n=61), 6 multi-case families (n=16)	5
Araújo et al	2012	Brazil	Cross-sectional	Household (1352)	Cases (n=444)	5
Sato et al	2022	Brazil	Cross-sectional	Social - schools (236)	.	6
Barreto et al	2021	Brazil	Case-control	Household (41) and peridomestic (40)	Cases (n=22)	5
Pathak et al	2019	India	Cross-sectional	Household (50)	Cases (n=91)	3
Carvalho et al	2018	Brazil	Cross-sectional	Household (48)	Cases (n=66) and Control (n=80)	4
Gama et al	2018	Brazil	Cross-sectional	Household (113)	Cases (n=43), negative controls (n=8)	4
Gama et al	2019	Brazil	Cohort	Household (2011:113) (2012: 80) (2016: 44)	Cases (n=38), Control (n=40)	6
Manta et al	2019	Brazil	Cohort	Household (980), and of these 955 were included in the cohort and underwent molecular evaluation for screening	.	7
Arunagiri et al	2017	India	Cross-sectional	Household (100)	No contacts (n=100)	3
Smith et al	2004	India	Cohort	Household (202), and of these 157 underwent molecular evaluation	No contacts (n=2,832)	5

*Newcastle-Ottawa Scale

Continue

Citation	Year of publication	Location (Country)	Study design	Sample - Type of Contact (n)	Other groups (n)	NOS*
Cardona-Castro et al	2008	Colombia	Cross-sectional	Household (402), and of these 71 underwent molecular evaluation	.	4
Brito e Cabral et al	2013	Brazil	Cross-sectional	Household (135)	Case (n=30)	4
Krismawati et al	2020	Indonesia	Cross-sectional	Household (107)	.	4
Guerrero et al	2002	Colombia	Cross-sectional	Household (70)	.	4
Ramaprasad et al	1997	India	Cohort	Household (134) and of these 106 underwent molecular evaluation and 73 participated in the cohort	Control - indigenous population (n=89), health workers (n= 33), cases (n= 48)	4
Job et al	2008	India	Cohort	Household (93)	Cases (n=20)	5
Reis et al	2014	Brazil	Cohort	Household (826)	Cases (n=200)	8
Martins et al	2010	Brazil	Cross-sectional	Household and peridomestic (31)	Control group (n=6): 5 negative for anti-PGL-1 and 1 positive	2

Supplementary Material Table S4 - Characteristics of molecular analyses and outcome

Citation	Molecular evaluation - Target	Biological material analyzed	Analysis technique	Outcome and main results
Turankar et al	Presence - RLEP	Dermal scraping of earlobes	Conventional PCR	*M. leprae* DNA positivity among contacts was 21.43% (6/28). Among the 5 cases evaluated, all showed amplification for RLEP. The authors also carried out subtyping of the *M. leprae* DNA identified in the samples, and from this they concluded that the source of infection was the associated index case, or that both had the same primary source of infection.
Cardona-Castro et al	Presence - RLEP	Dermal scraping of earlobes, lesions and elbows and nasal swab	Conventional PCR	*M. leprae* DNA positivity among contacts was (3.12%) 1/32. This same contact at the time of data collection had a mild, hypopigmented skin lesion (2 x 3 cm), located on the left thigh, with an anesthetic area around the lesion. After this contact with DNA from the identified bacillus, he developed PB leprosy. The authors also evaluated anti-PGL-1 serology and identified a seroconversion rate of 28% among the contacts.
Wen et al	Presence - RLEP	Blood	Conventional PCR	*M. leprae* DNA positivity among contacts was 6.25% (6/96) among healthy close contacts.
Almeida et al	Presence - RLEP	Blood and nasal swab	Conventional PCR	4/120 (3.4%) were positive for *M. leprae* DNA in blood or nasal secretions, of which 2/119 (1.7%) were positive for blood samples and 2/120 (1.7%) were positive for nasal swabs. None of the contacts fell ill during follow-up.

Continue

Citation	Molecular evaluation - Target	Biological material analyzed	Analysis technique	Outcome and main results
Banerjee et al	Presence - RLEP	Nasal swab	Conventional PCR	Positivity to *M. leprae* DNA was 7.14% (13/182), with 10.9% (12/110) among contacts of MB cases and 1.3% (1/72) among PB contacts. Positivity was higher among adults than among children. The 1.09% (2/182) of contacts who fell ill were MB cases.
Barreto	Presence - RLEP	Nasal swab	Conventional PCR	*M. leprae* DNA positivity was 22.75% (33/145). Among healthy contacts, 20.5% (26/127) tested positive. In the group of sick household contacts, 38.88% (7/18) tested positive. Among the 187 participating contacts, 11.8% (22/187) developed leprosy, 16 of whom became ill between the index case diagnosis and data collection and 6 of whom were diagnosed during data collection.
Lourenço et al	Presence - RLEP	Nasal swab	Conventional PCR	Positivity to *M. leprae* DNA was 16.1% (14/87). The percentage of contacts between the ages of 11 and 15 who tested positive in the PCR was significantly higher than the individuals of this age who tested negative (85.7% (12/14) vs. 26% (19/73); P<0.0001). With regard to the operational classification of the index case, the percentage of PCR positivity was 15.1% (8/53) among contacts of PB cases and 17.6% (6/34) for MB contacts, with no significant difference (p= 0.05).
Medina et al	Presence - RLEP	Nasal swab	Conventional PCR	19.23% (5/26) contacts were positive for *M. leprae* DNA. Of these, 4 were contacts of MB cases and 1 of a PB case.

Continue

Citation	Molecular evaluation - Target	Biological material analyzed	Analysis technique	Outcome and main results
Romero-Montoya et al	Presence - RLEP	Nasal swab	Conventional PCR	*M. leprae* DNA positivity was 16% (18/113). These 18 contacts belonged to 12 family groups, which indicates that two family groups had two contacts with *M. leprae* and one group had three contacts with *M. leprae*. 2 new cases of leprosy (1.76%) were confirmed among the contacts after the tests.
Silva et al	Presence - RLEP	Nasal swab	Conventional PCR	*M. leprae* DNA positivity was 72.2% (13/18) for the R5/R6 assay, with 100% (n=4) of PB case contacts and 64.29% (n=9) of MB case contacts being positive for this assay. For the LP1/LP2 test, 66.6% (12/18) were positive, with 100% (n=4) of PB case contacts and 57.14% (n=8) of MB case contacts testing positive.
Urgesa et al	Presence - RLEP	Nasal swab	Conventional PCR	Positivity to *M. leprae* DNA was 11.4% (5/44) (95%CI: 3%, 24%), and all contacts with positive PCR were contacts of MB patients. There was no significant difference in PCR positivity between the sexes (p = 0.622), age groups (P = 0.609) and duration of contact with the index case (p = 0.712).
Pinho et al	Presence - RLEP	Nasal and oral swab	Conventional PCR	Positivity to *M. leprae* DNA was 8.5% (69/808) of the samples from contacts. Of these, 5.6% (45/808) were oral swabs and 4.6% (37/808) were nasal swabs. Among the 69 contacts who tested positive, 21.7% (15/69) were positive in both samples.

Continue

Citation	Molecular evaluation - Target	Biological material analyzed	Analysis technique	Outcome and main results
Martinez et al	Presence - RLEP	Oral swab	Conventional PCR	The overall positivity for *M. leprae* DNA was 6.83% (88/1288). When assessing the contacts of PB and MB patients, 5.08% (15/295) and 7.35% (73/993) were positive, respectively, with no significant difference between the groups (X^2= 0.415; p=0.7187).
Tió-Coma et al	Presence - RLEP	Dermal scraping of earlobes and nasal swab	Conventional PCR and qPCR	*M. leprae* DNA positivity among contacts was 12.3% (30/250) in dermal scraping samples and 18.0% (45/250) in nasal *swabs*. The percentage of contacts who fell ill during follow-up was 1.6% (4/250). Of these, one had a positive PCR in a dermal scraping 5 months earlier and another in a nasal *swab* 8 months before diagnosis. The other two had positive anti-PGL-1 serology 10 and 12 months before diagnosis, so that all the new cases were positive for associated diagnoses 5 to 12 months before developing the disease. Individual anti-PGL-I levels showed a negative correlation with Ct RLEP values.

Continue

Citation	Molecular evaluation - Target	Biological material analyzed	Analysis technique	Outcome and main results
Araujo et al	Presence - RLEP	Nasal vestibule biopsy, nasal swab, blood	qPCR	*M. leprae* DNA positivity was 49% (51/104) for nasal swabs, 53.8% (56/104) for nasal concha biopsies and 6.7% (7/104) for blood samples. Among the 104 contacts, 6.7% (7/104) developed leprosy during the 5-7 year follow-up period. A significant relationship was identified between qPCR results for nasal swabs and turbinate biopsy samples (OR=2.3; 95%CI: 1.0-5.1), as well as between qPCR results for nasal turbinate samples and anti-PGL-I serology (OR=4.2; 95%CI: 1.2- 14.8; p= 0.046). There was a clear probability of developing leprosy among contacts who were positive for qPCR in blood samples (positive likelihood ratio (LR+) and relative risk (RR): 5.54 (95%CI: 1.30-23.62).
Bouth et al	Presence - RLEP	Dermal scraping of earlobes	qPCR	Positivity to *M. leprae* DNA was 23.7% (18/78). 2.6% of contacts were double positive (anti-PGL-I+/RLEP qPCR+). Illness was identified among contacts of treated cases: 5.3% (3/57); contacts of cases identified among schoolchildren: 17.6% (6/34); as well as among contacts of spontaneous demand cases: 10% (2/20).
da Silva et al	Presence - RLEP	Dermal scraping of earlobes	qPCR	Positivity to *M. leprae* DNA was 27.4% (81/296). The sensitivity of qPCR for DNA detection was 84%, specificity 75% and accuracy 77%. 15.5% (46/296) of contacts were positive for both RLEP and anti-PGL-1 serology.

Continua

Citation	Molecular evaluation - Target	Biological material analyzed	Analysis technique	Outcome and main results
Das et al	Presence - RLEP	Dermal scraping of earlobes	qPCR	*M. leprae* DNA positivity was 78.7% (48/61). Contacts of BL and LL cases were more positive for *M. leprae* DNA than tuberculoid cases (OR= 6.6; 95%CI: 1.6-27.6; p= 0.0090). Similarly, contacts of MB cases (OR: 4.88; 95%CI: 1.02-23.37; p= 0.04) and those with a positive baciloscopic index (BI) (OR: 7.07; 95%CI: 1.41-35.41; p= 0.0173) showed greater molecular positivity. Being a genetic contact of the index case also increased the chances of positivity when the case was LL or BL (OR: 9.23; 95%CI: 1.01-83.94; p= 0.04) and had a positive BI (OR: 6.93; 95%CI: 0.76-63.04; p= 0.08).
Araújo et al	Presence - RLEP	Nasal swab	qPCR	*M. leprae* DNA positivity was 4.7% (63/1,352), 3.3% (10/303) for contacts of PB cases and 5.1% (53/1,049) for contacts of MB cases. With regard to the classification of the clinical form of the index case, contacts were positive for *M. leprae* DNA: 0% (0/9) contacts of cases in the undetermined clinical form, 3.7% (4/107) TT, 3.9% (16/412) BT, 5.7% (14/244) BB, 7% (17/241) BL, 3.6% (12/339) LL. There was a significant association between PCR results using nasal swabs and anti-PGL-I serology results ($p < 0.0034$).

Continue

Citation	Molecular evaluation - Target	Biological material analyzed	Analysis technique	Outcome and main results
Sato et al	Presence - RLEP	Nasal swab	qPCR	Positivity to *M. leprae* DNA among social contacts was 14% (33/236), ranging from 5.1% to 50% between schools. There was a higher prevalence of social contacts with positive PCR among those living in the northern region of the city (42.4%), aged between 10 and 14 (72.7%) and belonging to socioeconomic classes C, D or E compared to the highest economic class (66.7%). In the bivariate analysis, there was a statistically significant difference between the variables region of residence in which living in the north, south and east had a higher chance of infection by *M. leprae* (PR: 0.40; 95% CI: 0.18-0.91; p=0.039) and condition of residence in which living in a rented or donated property had a higher chance of infection by *M. leprae* (PR: 2.16; 95% CI: 1.16-4.04; p= 0.015).
Barreto et al	Presence - RLEP and 16S rRNA	Saliva	Conventional PCR	In the group of household contacts alone, *M. leprae* DNA positivity was 4.9% (2/41) for RLEP and 2.4% (1/41) for 16S rRNA. With regard to peridomiciliary contacts, 12.5% (5/40) were positive for RLEP and 2.5% (1/40) for 16S rRNA. Considering the two groups as a single group of contacts, the positivity was 8.6% (7/81) for RLEP and 2.5% (2/81) for 16S.
Pathak et al	Presence - RLEP, 16SrRNA and sodA	Blood, nasal swab, dermal scraping from earlobes, saliva	Conventional PCR	In PCR analyses with individual genes, positivity for *M. leprae* DNA was 10% (5/50) for RLEP and 4% (2/50) for 16S. The SodA target was not positive. In M-PCR, the percentage of positives was 40% (20/50).

Continue

Citation	Molecular evaluation - Target	Biological material analyzed	Analysis technique	Outcome and main results
Carvalho et al	Presence - RLEP and 85B	Nasal swab and palate scraping	qPCR	Positivity to *M. leprae* DNA was 31% (15/48) in the palate mucosa and 38% (18/48) in the nasal mucosa for RLEP, with no significant difference (p= 0.6674). For target 85B, positivity was 13% (6/48) in the palate mucosa and 19% (9/48) in the nasal mucosa, also with no difference in positivity between collection sites (p= 0.5740).
Gama et al	Presence - 16S rRNA	Dermal scraping of earlobes and blood	qPCR	The overall positivity for *M. leprae* DNA was 23.89% (27/113), with 19.23% among PB contacts and 27.87% among MB contacts. Looking at the results separately, blood samples were 9.73% positive (11/113), 7.69% among PB contacts and 11.48% among MB contacts. For dermal scraping samples, positivity was 15.93% (18/113), 13.46% among contacts of PB cases and 18.03% among contacts of MB cases. After 1 year of follow-up, 2.65% (3/113) of the contacts were diagnosed with leprosy.
Gama et al	Presence - 16s rRNA	Dermal scraping of earlobes and elbows	qPCR	Positivity to *M. leprae* DNA was 16.81% (19/113) in 2011, 7.04% (5/71) in 2012, and 0% (0/46) in 2016. For contacts of PB cases, positivity was 11.5% (7/52) in 2011, 3.5% (1/29) in 2012 and 0% (0/25) in 2016. For contacts of MB cases, it was 19.7% (12/61) in 2011, 9.5% (4/42) in 2012 and 0% (0/21) in 2016. There was a reduction in the frequency of positive individuals during the study period. 4 contacts fell ill during follow-up, 3 in 2012 and 1 in 2017, but the relationship between the positive test results and the contacts who fell ill was not verified.

Continue

Citation	Molecular evaluation - Target	Biological material analyzed	Analysis technique	Outcome and main results
Manta et al	Presence - 16S rRNA	Dermal scraping of earlobes	qPCR	Positivity to *M. leprae* DNA was 9% (86/955). The number of contacts who fell ill was 0.5% (5/955). Among the contacts who became ill, 20% tested positive for qPCR, and among those who did not become ill, this positivity was 9%. There was a relative risk of 2.52 (95%CI: 0.28-22.35) for predicting leprosy, but this result was not statistically significant.
Arunagiri et al	Presence - to	Nasal swab	Conventional PCR	Positivity to *M. leprae* DNA was 13% (13/100).
Smith et al	Presence - to	Nasal swab	Conventional PCR	Positivity to *M. leprae* DNA was 0.6% (1/157). There was no significant association between *M. leprae* DNA positivity and being a household contact.
Cardona-Castro et al	Presence - 12-5	Nasal swab	Conventional PCR	Positivity to *M. leprae* DNA was 31% (22/71). Among the contacts who were PCR positive, 72.7% (16/22) were also positive for anti-PGL1.
Brito e Cabral et al	Presence - 36 kDa	Nasal swab	Conventional PCR	*M. leprae* DNA positivity was 26.7% (12/135). Of these, 3 were contacts of PB cases and 9 of MB cases. Among the 12 contacts positive for *M. leprae* DNA, 5 were positive for serum anti-PGL1 IgM, 7 were positive for salivary anti-PGL1 antibodies and 1 was positive for all the isotypes tested, with the exception of salivary anti-PGL1 IgM.

Continue

Citation	Molecular evaluation - Target	Biological material analyzed	Analysis technique	Outcome and main results
Krismawati et al	Presence - LP1 (5'-TGC ATGTCATGGCCTTG AGG-3') and LP2 (5'-CAC CGATACCAGCGGCA GAA-3')	Nasal swab	Conventional PCR	Positivity to *M. leprae* DNA was 19.62% (21/107). Contacts who had lived with the leprosy patient for more than 1 year had a 12 times greater risk of contracting *M. leprae* than those who lived away from the patient (OR= 12.45, 95%CI: 1.595-97.20; p=0.002).
Guerrero et al	Presence - LSR/A15	Nasal swab	Conventional PCR	Positivity to *M. leprae* DNA was 12.8% (9/70). Spouses were more likely to be infected than other cohabitants, regardless of relationship (OR=3.87, 95%CI: 1.21-12.3).
Ramaprasad et al	Presence - S13/S62	Nasal swab	Conventional PCR	Positivity to *M. leprae* DNA was 2% (2/106) in the first approach, and in the follow-up positivity rose to 4.1% (3/73), with the contact who was positive and took part in the follow-up having a negative PCR result and 3 others having a positive result.
Job et al	Presence - 18kDa	Dermal scraping of forearm and wrist and nasal swab	Conventional PCR	*M. leprae* DNA positivity was 17.2% (16/93) in dermal scraping samples before case treatment, and 1.07% (1/93) one month after case treatment. In nasal swab samples, positivity was 4.3% (4/93) before treatment of the case and 6.45% (6/93) one month after treatment of the index case. Of these 6, two were positive at both times. Two months after treatment of the index case, none of the contacts tested were positive in any type of sample.

Continue

Citation	Molecular evaluation - Target	Biological material analyzed	Analysis technique	Outcome and main results
Reis et al	Presence - ML0024	Blood	qPCR	Positivity to *M. leprae* DNA was 1.2% (10/826), with 1.1% (2/182) in contacts of PB patients and 1.2% (8/644) in contacts of MB patients. All household contacts with ML0024 positive qPCR (n=10) also had negative or weakly positive Mitsuda tests (p=0.0131) and 40% (4/10) were positive for anti-PGL-1 (p=0.1283). Contacts with the presence of *M. leprae* DNA had a higher chance of developing leprosy (OR: 14.78, 95%CI: 3.6-60.8; p<0.0001) and a positive likelihood ratio of 13.19 (95%CI: 3.6-48.1; p<0.0001). In a 7-year follow-up, 3.1% (26/826) of contacts developed leprosy. Among these 26 contacts, 11.5% (3/26) had a positive qPCR result.
Martins et al	Presence - Not mentioned	Nasal mucosa biopsy	qPCR	*M. leprae* DNA positivity was 19.35% (6/31). 3.22% (1/31) of the contacts identified in the year following the molecular evaluation were ill with leprosy. All 6 PCR-positive contacts had positive anti-PGL-1 serology results, with five of them having positive serum levels of 2+ and one having a serum level of 4+.
